# Development of a new metastatic human breast carcinoma xenograft line.

**DOI:** 10.1038/bjc.1998.96

**Published:** 1998-02

**Authors:** R. R. Mehta, J. M. Graves, A. Shilkaitis, T. K. Das Gupta

**Affiliations:** Department of Surgical Oncology, University of Illinois at Chicago, 60612, USA.

## Abstract

**Images:**


British Journal of Cancer (1998) 77(4), 595–604
© 1998 Cancer Research Campaign

### Development of a new metastatic human breast carcinoma xenograft line

**RR Mehta, JM Graves, A Shilkaitis and TK Das Gupta**

Department of Surgical Oncology, University of Illinois at Chicago, 840 South Wood Street, M/C 820, Chicago, IL 60612, USA

**Summary** Xenografts originated from human tumours offer the most appropriate research material for in vivo experimental research. However, primary human breast carcinomas are difficult to grow when transplanted in athymic mice: tumour take is less than 15%. Recently, we have achieved 60% tumour take by injecting tumour cell suspensions mixed with Matrigel. Human breast xenografts originated from primary breast carcinoma also frequently show the potential to metastasize spontaneously. In the present study, we generated a human breast carcinoma xenograft line (UISO-BCA-NMT-18) that shows 100% tumorigenicity and 80–100% lung metastasis when transplanted s.c. in athymic mice. We have studied in detail the characteristics of the xenograft and the patient's tumour from which the xenograft line originated. Both the xenograft and the patient's tumour showed intense staining for mutant p53 nuclear protein, and high expression of U-PA, PAI and u-PAR. In vivo growth of the xenograft is stimulated by exogenous supplementation of oestrogen. This xenograft is continuously growing in mice and has shown 80–100% metastasis for the last three successive in vivo passages. This well-characterized, oestrogen-responsive, metastatic breast carcinoma xenograft line will provide excellent research material for metastasis-related research.

**Keywords:** breast cancer; xenograft; cell line; metastasis

Metastasis of primary tumour to the visceral organs is a major cause of cancer-associated mortality. Despite considerable progress in the last decade in the areas of early detection and surgical and chemotherapeutic management of clinically less advanced cancers, treatment of metastatic disease is still a major puzzle for oncologists. Research on metastatic disease has severely suffered from lack of suitable experimental models. Human tumour xenografts established from well-characterized clinical material provide an important research tool for multidisciplinary research. In most cases, xenografts originated in vivo in experimental animals preserve many of the original phenotypic, biological and genotypic characteristics from which they originate (Giovanella et al, 1989; Mehta et al, 1995*a*). However, human breast carcinomas are difficult to grow in vitro in culture (Nordquist et al, 1975; Engel and Young, 1978; Langlois et al, 1979; Whitehead et al, 1983; Chu et al, 1985; Mehta et al, 1992; 1995*b* ; Watanabe et al, 1992) or in vivo in experimental animals (Sabestany et al, 1979; Shafie and Liotta, 1980; Rae-Venter and Reid, 1980; Giovanella et al, 1985; Price and Zhang, 1990; Hurst J, et al, 1993; Mehta et al, 1993), and they rarely metastasize when transplanted subcutaneously (Shafie and Liotta, 1980; Price et al, 1990; Brunner et al, 1992; Mehta et al, 1993).

Recently, we obtained significant success in establishing xenografts from primary breast carcinomas, and many of these xenografts showed potential for spontaneous metastasis in athymic mice when transplanted s.c. (Mehta et al, 1993). Initially, we used Matrigel to develop xenografts from primary breast carcinomas (Mehta et al, 1993; 1995a). Matrigel is a mixture of components usually found in the extracellular matrix. The major components of Matrigel are laminin, collagen IV, heparan sulphate and entactin. Matrigel not only increased the take rate of human breast carcinoma in athymic mice, but also promoted spontaneous metastasis in approximately 40% of tumours (Mehta et al, 1993). In the present study, we further explored the metastatic potential of a xenograft by serial repetitive in vivo/in vitro propagation of a human breast xenograft originated in mice. We have established a human breast carcinoma xenograft line that is 80–100% metastatic when transplanted s.c. in athymic mice.

*Received 29 November 1996*
*Revised 26 June 1997*
*Accepted 8 July 1997*

*Correpondence to:* RR Mehta

### MATERIAL AND METHODS

### Procurement of human breast carcinoma

Primary human breast carcinoma was obtained from a 41-year-old woman with confirmed diagnosis of primary human breast carcinoma undergoing lumpectomy procedure. After surgical excision, tumour tissue was immediately transported to the laboratory on wet ice. Detailed information regarding histopathological details of the tumour and the patient's disease status was obtained from our tumour registry.

### Enzymatic digestion of tumour

Tumour tissue was divided into small pieces, one of which was fixed in 10% formalin and processed for histological and immunohistochemical analysis of various biomarkers. The remaining tissue (approximately 0.2–0.3 g) was minced into small pieces and mixed (1:10 volume) with a cocktail of enzymes composed of 0.002% deoxyribose nuclease type 1 (Sigma, St Louis, MO, USA), 0.1% collagenase (United States Biochemical Corporation, Cleveland, OH, USA) and 0.01% hyaluronidase type V (Sigma, St Louis, MO, USA) in Hanks' balanced salt solution (HBSS, Biologos, Naperville, IL, USA), then incubated overnight at room temperature. At the end of incubation, enzyme-digested tissue

A B C D E F

© Cancer Research Campaign 1998

**Figure 1** Patient's original tumour. (**A**) Histology of the tumour; (**B**) mutant type p53 protein; (**C**) immunohistochemical detection of oestrogen receptor; (**D**) immunohistochemical detection of progesterone receptor; (**E**) nm23 protein; (**F**) cathepsin D; (**G**) Her-2/neu; (**H**) urokinase-type plasminogen activator; (**I**) plasminogen activator receptor; (**J**) plasminogen activator inhibitor-1

suspension was centrifuged, and the tissue pellet was rinsed with HBSS then suspended in HBSS/Matrigel mixture (1:1 volume) (Collaborative Biomedical Products, Becton Dickinson Labware, Bedford, MA, USA).

The tumour suspension was injected into the mammary fat pad of 3- to 4-week-old female athymic (Balb/c) mice (Frederick Cancer Research Facility, Frederick, MD, USA). Animals were observed for development of a palpable tumour; if a tumour developed the tumour size (cm) was measured in three different planes as height ($h$), width ($w$) and depth ($d$), using vernier calipers.

Tumour volume (cm$^3$) was calculated using the formula $h \times w \times d \times \pi/6$ = cm$^3$. For this study, the tumour doubling time was calculated as the number of days required during the exponential growth phase for the tumour to grow from $x$ volume to $2x$ volume. The tumour latency period is the time (days) required for the tumour to show apparent sustained increase in volume from the initial volume of injected suspension or xenograft.

Animals were killed if they became moribund, had necrosis in the tumour, or if the tumour volume reached > 2.5 cm$^3$ in volume. All animals at termination were examined for metastatic lesions in the visceral organs. Suspected lesions were processed for histopathological examinations. The tumour that developed at the inoculation site was divided into small pieces and processed for histological studies, further in vivo propagation and studies of various biochemical and immunohistochemical biomarkers.

### Serial in vivo processing of human tumour xenograft in athymic mice

The xenograft developed at the original inoculation site was divided into small pieces and then trocared s.c. into athymic mice. Matrigel was not used for serial transplantation of xenografts. Tumour that developed during each serial passage was processed

© Cancer Research Campaign 1998

for histology and lactose denydrogenase (LDH) isoenzyme pattern to confirm human origin of the xenograft.

### Effect of low-dose oestrogen on in vivo growth of xenografts

To determine the effect of oestrogen on the growth of xenografts, xenografts were transplanted s.c. in the dorsal flank of 3- to 4-week-old athymic mice. Animals received a s.c. oestradiol-17β (0.1 mg per animal)-containing pellet (Innovative Research, Toledo, OH, USA) or placebo pellet. The growth of xenografts was monitored periodically as described above.

### Development of a metastatic tumour line by in vivo/in vitro propagation of metastatic lesion

The xenografts were serially passaged in vivo in athymic mice as mentioned above. If animals showed tumour metastasis in the lung, metastatic tumour was transplanted s.c. into 4- to 6-week-old female athymic mice. The tumour that developed at the subcutaneous site was passaged twice in vivo then cultured in vitro, and cells growing in vitro were mixed with Matrigel and then injected into mice again. Tumour that developed from the cultured cells was further passaged in vivo.

### LDH isoenzyme analysis

LDH isoenzyme pattern in human breast xenograft developed in mice was examined using the kits obtained from Corning Scientific Products, Corning, NY, USA. Known human cell lines were included as controls in each analysis.

### Immunohistochemical analyses of various proteins

Immunohistochemical analyses of various protein biomarkers in the original breast tumour and in the xenograft developed in mice were performed by the indirect immunoperoxidase method, with a labelled streptavidin–biotin complex kit (Dako Corporation, Carpinteria, CA, USA). In brief, 4- to 5-μm-thick sections of formalin-fixed paraffin-embedded tissues were mounted on frosted microslides, and sections were deparaffinized in xylene and rehydrated by processing through a graded series of alcohol

**Figure 2** Light microscopic examination of human breast carcinoma UISO-BCA-NMT-18; (**A**) Xenograft from in vivo passage 1; (**B**) xenograft from passage 2; (**C**) xenograft from passage 7.

**Figure 3** Immunohistochemical detection of (**A**) p53 protein and (**B**) Her-2/neu in UISO-BCA-NMT-18 xenograft

© Cancer Research Campaign 1998

**Figure 4** (**A** and **B**) Western blot analyses of Her-2/neu and nm-23 in UISO-BCA-NMT-18 xenograft (at passage 2/3) extract. Arrow shows protein band of interest. M, Molecular weight markers

(100–0%). The tissue sections were rinsed in phosphate-buffered saline (PBS) and then microwaved three times for 5 min in citrate buffer (c-neu, Cathepsin) or in 6 M urea (p53) at 80% power. For U-PA, PAI-1 and UPAR studies, rehydrated tissue sections were incubated for 30 min in 0.1% trypsin at 37°C. The sections were extensively washed with PBS.

To block non-specific binding, sections were incubated at room temperature in 5% non-fat dry milk for 10 min. Tissue sections were rinsed in PBS and then incubated at 4°C overnight in the moisture chambers with appropriate diluted specific primary antibody. Tissue that had been incubated with mouse IgG (5 μg $ml^{-1}$) served as an experimental control. At the end of the incubation, sections were extensively rinsed in PBS, then incubated with biotinylated anti-mouse/anti-rabbit link antibody for 10 min and with peroxidase-conjugated streptavidin for 10 min. Staining was visualized using 3-amino 9-ethyl carbazole (AEC) or 3,3′-diaminobenzidine as chromogen (Biogenex, San Ramon, CA, USA). Tissues were counterstained with haematoxylin. The tissue sections were mounted in aqueous mounting medium. Primary antibodies were obtained from different suppliers. Antibodies for oestrogen receptor–progesterone receptor (ER/PR) were purchased from Abbott Laboratories, Lake Forest, IL, USA; p53 and Her-2/neu (Oncogene Science, Uniondale, NY, USA), nm-23 and cathepsin D from Neomarkers (Fremont, CA, USA); U-PA, UPAR and PAI from American Diagnostics (Greenwich, CT, USA).

### Western blot analyses

Western blot analyses of nm23 and Her-2/neu proteins were performed according to the method reported previously (Mehta et al, 1995*b*). In brief, cells were washed with PBS and lysed in Tris buffer, pH 6.8, containing 10% glycerol, 5% β-mercaptoethanol, 3% sodium dodecyl sulphate (SDS), 0.02% bromophenol blue, 1 mM phenylmethylsulphonyl fluoride, 5 mM EDTA, 10 μg $ml^{-1}$ aprotinin, and 1 μg $ml^{-1}$ DNAase. Cell lysis was performed at 37°C for 30 min. Proteins in the lysates were separated on 7.5% SDS-polyacrylamide gel by electrophoresis. Proteins were electroblotted to immunobilon paper overnight. The membrane was incubated with primary antibody (1 μg $ml^{-1}$) at room temperature for 1 h, washed with buffer, then incubated with goat anti-mouse alkaline phosphatase. Specific immunoreactivity was visualized using fuchsin as a chromogen.

### RESULTS

### Characteristics of human breast carcinoma

Histopathologically, the original patient's tumour was classified as infiltrating ductal carcinoma of the breast (Figure 1A). At the time of surgery, no evidence of disease having spread to the axillary lymph nodes was reported. The tumour was immunohistochemically positive for ER and PR and strongly positive for p53 and intensely stained for nm23. The tumour showed weak to moderate cytoplasmic staining for cathepsin D, strong cell membrane-associated staining for PAI, U-PAR and U-PA. The tumour showed strong immunoreactivity to Her-2/neu antibody (mouse monoclonal antibody against a specific peptide sequence from the

**Figure 5** Immunohistochemical detection of (**A**) U-PA, (**B**) U-PAI and (**C**) UPAR in UISO-NMT-BCA-18 xenograft

**Figure 6** Growth of UISO-BCA-NMT-18 xenograft in athymic mice during serial in vivo propagation. Number in parenthesis indicates passage number in vivo. Number of animals at each passage varied between 2 and 5, depending on the availability of xenograft material available. Data represent mean tumour volume value obtained in the number of animals used for that passage

**Figure 7** In vivo growth pattern of metastaic xenograft line. Data represent mean tumour volume obtained in a group of five animals that received xenograft transplant from the same tumour. Met-1, Met-2, Met-3 indicates metastatic passage number carboxyl domain of human Her-2 gene product); however, staining was predominantly localized in the cell membrane (Figures 1B–J).

### Growth of human breast carcinoma transplanted into athymic mice

The cell suspension injected into mice initially formed a small palpable tumour nodule 17 days after inoculation. The tumour nodule failed to show continued growth and remained as a small

**Figure 8** Growth of UISO-BCA-NMT-18 xenografts with low metastatic potential in athymic mice nodule for 60 days. The nodule was excised and retransplanted into two mice; at this time, two out of two animals formed tumours.

The tumour latency period in this passage was 20 days. The tumour doubling time at passage 1 was between 5 and 8 days. The tumour was hard and localized at the inoculation site. During successive serial passaging, 100% tumour formation was observed in animals. The xenografts at each in vivo passaging were confirmed to have human origin by LDH isoenzyme assay (data not shown).

### Characterization of human breast xenograft

Light microscopy of the xenograft at passage 1 showed small clusters of human breast carcinoma cells embedded in host stromal tissue. At passage 2, cell clusters grew more compact and had minimal presence of host stromal tissue elements. The xenograft from which 100% lung metastasis was first observed (passage 7) showed histopathology similar to that of the original patient's tumour. The clusters of tumour cells were infiltrated in host stromal tissue, and evidence was frequently observed of necrotic tissue scattered throughout the tumour tissue. At this time, many tumour cells were seen at the different phases of cell division (Figure 2A–C).

Immunohistochemical analyses of various biomarkers were performed on the xenograft at in vivo passages 2 and 3 (before generating the metastatic line). Immunohistochemically, detectable specific staining was observed against Her-2/neu, p53 (Figure 3A–B), and nm23 (data not shown). The presence of Her-2/neu protein was further confirmed by Western blot analysis of xenograft extract (Figure 4A). Because nm23 antibody used in the present study cross-reacts with two different proteins (nm23-H1 and nm23-H2), we performed Western blot analysis on xenograft lysate. On the SDS gel using denaturing condition in the cytosolic extract, we observed two specific proteins immunoreactive to nm23 antibody, approximately molecular weight 17 000 and 18 000 representing both nm23-H1 and nm23-H2 proteins (Figure 4B).

© Cancer Research Campaign 1998

**Figure 9** Histology of representative metastatic lesions in lungs. Size and number of metastatic lesions varied from animal to animal

We failed to observe specific immunoreactivity to antibodies against ER, PR or cathepsin D in the xenograft. We observed mild to moderate membrane-associated staining in the xenograft for U-PA, UPAI and UPAR (Figure 5A–C).

### Development of metastatic human breast xenograft line

The growth pattern of the xenograft during passage 1–7 shows that, at early passages, growth was initially slow; however, after passage 5, growth was enhanced. Initially, during passages 1–4, the tumour showed longer tumour latency time and failed to achieve true exponential growth. After passage 5, the tumour began to show increased growth between 13–20 days; tumour doubling time was between 8 and 9 days (Figure 6). Human breast xenograft serially transplanted for six passages in athymic mice showed no evident metastasis to visceral sites at any passages. However, in the seventh passage, one out of three animals had lung metastasis. At this time, the lung lesion was transplanted s.c. in one animal to expand the tumour material, and the tumour that developed at the injection site was passaged in two animals. The xenografts developed in these animals were minced, and a small portion was cultured in vitro and the remaining tissues were retransplanted in mice and serially passaged.

The cells growing in culture from xenograft were transplanted s.c. in two animals. At this time, one out of two animals showed metastatic tumour in the lung. The xenograft developed at the site of inoculation from the later animal was transplanted into five animals. All five animals developed tumours and attained experimental growth phase within 20–25 days; the tumour doubling time in these animals was between 10 and 12 days. All five animals showed metastasis in the lung (Met-1). We excised the xenografts growing at the inoculation site from this last group of animals and transplanted them into 15 animals. All 15 animals had tumours growing at the inoculation site (Met-2) within 17–20 days, and 13 out of 15 animals had lung metastasis. In the next transplantation (Met-3) into the animals, lung metastasis was found in 13 out of 14 animals. Figure 7 shows in vivo growth of xenografts with >80% metastatic potential in vivo.

The xenografts serially passaged (to passage 12) in vivo without in vitro propagation were 100% tumorigenic and showed occasional incidence (10–20%) of lung metastasis. Detailed analysis of growth pattern showed that, in general, xenografts developed in these animals had a relatively longer tumour doubling time (ranging between 15 and 18 days, mean tumour doubling time = 16.8 + 0.9 days) compared with those xenografts with higher metastatic ability. Figure 8 shows the growth of xenografts during serial in vivo transplantation.

**Figure 10** Effect of oestrogen supplement on in vivo growth of UISO-BCA-NMT-18 xenograft. The xenograft (Met-3) was minced into small pieces and transplanted (without Matrigel) s.c. into the dorsal flank region of 4- to 6-week-old female athymic mice. Animals were divided into two groups, each group consisting of five animals: (a) receiving placebo pellet, and (b) receiving oestradiol-17β (0.1 mg)-containing pellet. Data represent mean tumour volume obtained in five animals. Animals were killed when animals in control group appeared sick. At autopsy, all visceral organs were examined for evidence of metastasis

Figure 9 shows representative metastatic lesions developed in mice. The number and volume or size of metastatic tumours in the lungs varied widely from animal to animal and in vivo passage to passage. Occasionally, we observed multiple metastatic tumours in both lungs; however, most animals had 1–2 lesions per lung. Histologically, lesions formed in the lungs were identical to the xenograft developed at the subcutaneous site.

### Growth response to exogenous oestrogen

To determine whether UISO-BCA-NMT-18 xenografts (Met-3) have maintained functional ER, we determined the response of exogenously supplemented oestradiol on growth of these tumours. As shown in Figure 10, growth of xenografts transplanted without Matrigel into athymic mice bearing oestradiol pellets (0.1 mg) was significantly ($P < 0.05$) higher than those transplanted into control animals with placebo pellets. Interestingly, at the termination of the experiment, five out of five mice treated with placebo pellets showed metastatic lesions in the lungs; however, zero out of five animals treated with oestradiol showed lung metastasis.

### DISCUSSION

Breast cancer is the most common cancer among women and the second leading cause of cancer-related death in women. Although significant progress has been made in the last decade for early detection of tumours and treatment of clinically less advanced carcinoma, management of advanced breast cancer has still not improved. The process of metastasis is complex: it involves cascades of various biochemical and molecular steps (Liotta et al, 1983; Nicolson, 1988). Factors associated with both the host tissue and the malignant cells play crucial roles in the invasion and metastasis of tumour cells (Boghaert et al, 1992; Lester and McCarthy, 1992). Even though various investigators are engaged in understanding the actual molecular and biological steps in the process of metastasis, the exact mechanism of the process is still not fully understood. In addition, research on evaluating new antimetastatic drugs for breast cancer is severely hampered because of the unavailability of suitable experimental models for breast cancer metastasis.

For experimental research on metastatic disease, a reliable experimental tool is necessary. At present, numerous breast carcinoma cell lines established from primary solid human breast carcinoma or from the metastatic pleural fluids are available for various research (Nordquist et al, 1975; Engel and Young, 1978; Langlois et al, 1979; Whitehead et al, 1983; Chu et al, 1985; Mehta et al, 1992; 1995b; Watanabe et al, 1992; Slooten et al, 1995); however, only a limited number of these cell lines are tumorigenic in mice, and only two to three of these cell lines show distant metastasis when transplanted s.c. into athymic mice (Shafie and Liotta, 1980; Price et al, 1990; Brunner et al, 1992; Mehta et al, 1993). In addition the incidence of metastasis in these cell lines varies in different laboratories (Shafie and Liotta, 1980). Thus, establishment of a well-characterized human breast carcinoma xenograft line with highly tumorigenic and metastatic potential in experimental animals is of great value in metastatic research.

In general, human breast carcinomas fail to grow when transplanted into athymic mice. The tumour take of human breast tumours is generally about 6–15% (Shafie and Liotta, 1980; Mehta et al, 1993). In our laboratory, tumour take is generally >60% when enzymically digested tumours are injected into mice mixed with Matrigel (Mehta et al, 1993; 1995*a*). Matrigel not only increased tumour take but also enhanced tumour growth and facilitated spontaneous distant metastasis (Mehta et al, 1993). In the present study, we have established a human breast carcinoma xenograft line from a primary human breast carcinoma using Matrigel as described previously (Mehta et al, 1993; Mehta et al, 1995a;).

To establish a xenograft in athymic mice, the original patient's tumour was digested with a cocktail of enzymes, and the resulting cell suspension was pelleted and mixed with Matrigel then injected into athymic mice. Initially, during the first in vivo passage, the tumour grew as a small nodule at the site of the original tumour inoculation. However, in subsequent serial passaging, 100% of animals showed tumour growth at the site of the original xenograft transplantation. During serial passaging, inconsistent but occasional incidence of lung metastasis was observed in animals, suggesting metastatic potential of the xenograft. We expanded the metastatic tumour cells in the xenograft by in vivo passaging of lung lesions. We further cultured the metastatic tumour in vitro and then inoculated the culture in vivo. Further continuous in vivo passaging of breast xenograft developed from a metastatic lesion generated a xenograft line that is 80–100% tumorigenic in athymic mice for the last three successive in vivo passages. The xenografts continuously passaged in vivo without in vitro exposure have maintained low metastatic potential. From our results it is evident that during in vitro culturing of a xenograft originating from a metastatic lesion, the selection of highly aggressive and metastatic cells occurs.

It appears that, during serial in vivo passaging, gradual changes in xenograft histopathology occurred. Initially, at passage 1, the xenograft showed small clusters of cells embedded in host stromal tissues. In passage 2 and thereafter, most of the tumour was packed with malignant breast cells with minimal presence of the host stromal tissue. In later passages, the xenograft histology appeared to be similar to that of human breast tumour – that is tumour cells were infiltrated in the host stroma as clusters of cells simulating infiltrating ductal carcinoma histopathology.

The xenograft UISO-NMT-BCA-18 preserved many phenotypic characteristics of the patient's tumour from which it originated. The original patient's tumour was positive for mutant type p53, a tumour-suppressor nuclear phosphoprotein. The patient's tumour also had high expression of U-PA, PAI and UPAR. All these markers are associated with highly aggressive breast cancer (Slamon et al, 1987; Berger et al, 1988; Clark and McGuire 1991; Allred et al, 1992; Caleffi et al, 1994; Gasparini et al, 1994; McDonald et al, 1995; Hamby et al, 1996). We observed intense immunoreactivity against antibodies to human Her-2/neu in the patient's tumour. A similar staining pattern was also evident in the xenograft. We further confirmed the presence of overexpressed c-*erb*B2 in xenografts using Western blot analysis. More recently, two additional genes called *nm23-1* and *nm23-2* are thought to influence the metastatic potential of malignant tumours (McDonald et al, 1995; Hamby et al, 1996). Experimental evidence suggests that altered expression of nm23-1 or nm23-2 protein is associated with increased metastatic potential (McDonald et al, 1995; Hamby et al, 1996). The patient's original tumour showed enhanced expression of nm23 protein immunohistochemically. The antibody used in our assay detects both nm23-1 and nm23-2 proteins. Thus, enhanced nm23 expression observed in the patient's tumour is probably the result of altered levels of nm23-2 protein levels compared with nm23-1; using Western blot analysis, we detected high levels of nm23-1 protein in the xenograft at early passages. Similarly, U-PA, PAI and UPAR have been shown to have prognostic significance (Janicke et al, 1993; Bouchet et al, 1994; Duffy et al, 1994; Foekens et al, 1994; Foekens et al, 1995). Thus, this xenograft line is ideal for evaluating new chemotherapeutic agents that will effectively prevent metastasis of highly aggressive tumours.

The patient's tumour had positive immunoreactivity for ER, PR and cathepsin D. In the xenograft at passages 2 and 3, we failed to

 © Cancer Research Campaign 1998

detect ER by immunohistochemical assay. However, increased growth response to exogenously supplemented oestrogen observed in later passages in the line with metastatic ability compared with the placebo control group suggests that these tumour lines have maintained functional ER status. Failure to detect ER in xenograft tumours could be due to down-regulation of this protein by endogenous ligands (in mice) of c-erbB2. Two different ligands, gp30 and p75, have been shown to down-regulate in a dose-dependent manner the expression of ER in ER positive BT-474 and MCF-7 breast carcinoma cell lines in oestrogen-depleted medium (Grunt et al, 1995). On the contrary, low levels of oestrogen (0.1–1 nM) treatment to oestrogen-responsive MCF cells have been reported to cause a rapid but sustained drop in Her-2/neu mRNA (Read et al, 1990), suggesting that oestrogen has a differential effect on the markers associated with tumour aggressiveness and cell proliferation. Our results on in vivo growth and metastatic behaviour of UISO-NMT-BCA-8 in mice in the presence/absence of oestrogen are in agreement with those obtained in vitro in human breast cancer cells by Read and associates (1990). In athymic mice, UISO-BCA-NMT-18 showed increased growth by oestradiol but showed inhibition of metastatic potential compared with untreated controls. In this tumour line, down-regulation of Her-2/neu expression by oestradiol may inhibit tumour metastasis. Further detailed studies are currently in progress to understand the mechanism of oestradiol action on various molecular markers associated with breast carcinoma cell proliferation and metastasis.

In summary, the human breast xenograft reported in the present study is of great value for human breast cancer research. Currently, for metastatic breast cancer research MDA-MB-231 and MDA-MB-435 are widely used (Brunner et al, 1992; 1993). Both these cell lines were originated from metastatic pleural effusion, are highly tumorigenic, and have high metastatic potential. In addition, both these cell lines have been reported to be ER-negative. To the best of our knowledge, no reliable, highly metastatic ER-positive cell line or xenograft line is available for research. In general, the UISO-NMT-BCA-18 xenograft line differs from the existing metastatic lines as it originated from primary human breast carcinoma and is responsive to oestrogen. Thus, the addition of a well-characterized ER-positive tumour line to an existing panel of metastatic human breast carcinoma cell lines will provide a valuable tool with which to study the role of various oestrogen-regulated genes in metastasis of human breast cancer.

### ACKNOWLEDGEMENTS

The authors wish to thank the Ladies Auxiliary of the West Side VA Hospital, NCI contract NO1-CO-74102/3S-1258, and the Department of Surgical Oncology Research Fund (various donors) for their generous support and Kevin Grandfield for his editorial expertise.

### REFERENCES

Allred DC, Clark GM, Tandon AK, Molina R, Tormey DC and Osborne CK (1992) Her-2/neu in node negative breast cancer: Prognostic significance of overexpression influenced by the presence of in situ carcinoma. *J Clin Oncol* **10:** 599–605

Berger MS, Locher GW, Saurer S, Gullick WJ, Waterfield MD, Groner B and Haynes N (1988) Correlation of c-erbB2 gene amplification and protein expression in human breast carcinoma with nodal status and nuclear grading. *Cancer Res* **48:** 1238–1243

Boghaert ER, Simpson JF and Zimmer SG (1992) Invasion in vitro of malignant Hamster brain tumor cells is influenced by the number of cells and the mode of malignant progression. *Invas Metastas* **12:** 12–23

Bouchet C, Spyratos F, Martin PM, Hacene K, Gentile A and Oglobine J (1994) Prognostic value of urokinase type plasminogen activator (UPA) and plasminogen activator inhibitors PAI-1 and PAI-2 in breast carcinoma. *Br J Cancer* **69:** 398–405

Brunner N, Boysen B, Romer J and Spang-Thomsen M (1993) The nude mouse as an in vivo model for human breast cancer invasion and metastasis. *Breast Cancer Res Treat* **24:** 257–264

Caleffi M, Teague MW, Jenson RA, Vnencak-Jones CL, Dupont WD and Parl FF (1994) P53 gene mutation and steroid receptor status in breast cancer. *Cancer* **73:** 2147–2156

Chu MY, Hagertty MG, Wiemann MC, Tibbetts LM, Sato S, Cimminings FJ, Bogaara HA, Leduc EH and Calabresi P (1985) Differential characteristics of two newly established human breast carcinoma cell lines. *Cancer Res* **45:** 1357–1366

Clark GM and McGuire WL (1991) Follow-up study of Her-2/neu amplification in primary breast cancer. *Cancer Res* **51:** 944–948

Duffy MJ, Reilly D, McDermott E, O'Higgins N, Fennelly JJ and Andreasen PA (1994) Urokinase plasminogen activator as a prognostic marker in different subgroups of patients with breast cancer. *Cancer* **74:** 2276–2280

Engel LW and Young NA (1978) Human breast carcinoma cells in continuous culture: A review. *Cancer Res* **38:** 4327–4339

Foekens JA, Schmitt M, Vanputten WL, Peters HA, Kramer MD, Janicke I and Klijn JGM (1994) Plasminogen activator inhibitor-1 and prognosis in primary breast cancer. *J Clin Oncol* **12:** 1648–1658

Foekens JA, Look MP, Peters HA, Van Putten WLJ, Portengen H and Klijn JGM (1995) Urokinase type plasminogen activator and its inhibitor PAI-1. Predictor of poor response to tamoxifen therapy in recurrent breast cancer. *J Natl Cancer Inst* **87:** 751–756

Gasparini G, Weidner N, Bevilacqua P, Maluta S, Dalla Palma P, Caffo O, Barbareschi M, Boracchi P, Marubini E and Pozza F (1994) Tumor microvessel density, P53 expression, tumor size, and peritoneal lymphatic vessel invasion are relevant prognostic markers in node-negative breast carcinoma. *J Clin Oncol* **12:** 454–466

Giovanella BC and Fogh J (1985) The nude mouse in cancer research. *Adv Cancer Res* **44:** 69–120

Giovanella BC, Vardeman DM, William LJ, Taylor DJ, Stehlin JS, Ullrich A, Gary HE and Slamon DJ (1989) Poor prognosis of breast cancer patients whose tumor took in mice. Correlation with amplification and overexpression of the Her-2/neu oncogene. *Proc Am Assoc Cancer Res* **30:** 60

Grunt TW, Saceda M, Martin MB, Lupu R, Dittrich E, Krupitza G, Harant H, Huber H and Dittrich C (1995) Bi-directional interactions between the estrogen receptor and the cerbB-2 signaling pathways: Heregulin inhibits estrogenic effects in breast cancer cells. *Int J Cancer* **63:** 560–567

Hamby CV, Mendola CE, Abbi A, Thomson J and Backer JM (1996) Overexpression of catalytically inactive NDPK-B/nm 23-2 suppresses the metastatic potential of line IV CL-1 human melanoma cells in nude mice. *Proc Am Assoc Cancer Res* **37:** 78

Hurst J, Maniar N, Tombarkiewicz J, Lucas F, Roberson C, Steplewski Z, James W and Perras J (1993) A novel model of a metastatic human breast tumor xenograft line. *Br J Cancer* **68:** 274–276

Janicke F, Schmitt M, Pache L, Ulm K, Harbeck N, Hofler H and Graef H (1993) Urokinase (U-pA) and its inhibitor PAI-1 are strong and independent prognostic factors in node negative breast cancer. *Breast Cancer Res Treat* **24:** 195–208

Langlois AJ, Holder WD, Iglehart JD, Nelson Rees WA, Wells SA and Bolognesi DP (1979) Morphological and biochemical properties of a new human breast cancer cell line. *Cancer Res* **39:** 2604–2613

Lester BR and McCarthy JB (1992) Tumor cell adhesion to the extracellular matrix and signal transduction mechanisms implicated in tumor cell motility, invasion and metastasis. *Cancer Metastas Rev* **11:** 31–34

Liotta LA, Rao CN and Barsky SH (1983) Tumor invasion and the extracellular matrix. *Lab Invest* **49:** 636–649

McDonald NJ, De la Rosa A and Steeg PS (1995) The potential roles of nm-23 in cancer metastasis and cellular differentiation. *Eur J Cancer* **31A:** 1096–1100

Mehta RR, Bratescu L, Graves JM, Hart GD, Shilkaitis A, Green A, Beattie CW and Das Gupta TK (1992) Human breast carcinoma cell lines: Ultrastructural genotypic and immunocytochemical characterization. *Anticancer Res* **12:** 683–692

Mehta RR, Graves JM, Hart GD, Shilkaitis A and Das Gupta TK (1993) Growth and metastasis of human breast carcinomas with matrigel in athymic mice. *Brest Cancer Res Treat* **25:** 65–71

© Cancer Research Campaign 1998

Mehta RR, Graves JM, Warso MA and Das Gupta TK (1995*a*) Overexpression of mutant P53 and c-erbB2 proteins and breast tumour take in mice. *Br J Cancer* **72:** 1160–1164

Mehta RR, Graves JM, Shilkaitis A, Hart GD and Das Gupta TK (1995*b*). Breast carcinoma cell line with metastatic potential in mice. *Intl J Oncol* **6:** 731–736

Nicolson GL (1988) Cancer metastasis: tumor cell and host organ properties important in metastasis to specific secondary sites. *Biochim Biophys Acta* **948:** 175–224

Nordquist RE, Ishmael DR and Lovig GA (1975) The tissue culture and morphology of human breast tumor line BOT-2. *Cancer Res* **35:** 3100–3105

Price JE and Zhang RD (1990) Studies of human breast cancer metastasis using nude mice. *Cancer Metastas Rev* **8:** 285–297

Rae-Venter B and Reid LM (1980) Growth of human breast carcinomas transplanted in nude mice and subsequent establishment in tissue culture. *Cancer Res* **40:** 95–100

Read LD, Keith D Jr, Slamon DJ and Katzenellenbogen BS (1990) Hormonal modulation of Her-2/neu protooncogene messenger ribonucleic acid and p185 protein expression in human breast cancer cell lines. *Cancer Res* **50:** 3947–3951

Sabestany A, Taylor-Papadimitriou J, Ceriani R, Millis R, Schmitt C and Trevan D (1979) Primary human breast carcinomas transplanted in the nude mice. *J Natl Cancer Inst* **63:** 1331–1337

Shafie SM and Liotta LA (1980) Formation of metastasis by human breast carcinoma cells (MCF-7) in nude mice. *Cancer Lett* **11:** 81–87

Slamon DJ, Clark GM, Wong SG, Lewvin WJ, Ullrich A and McGuire WL (1987) Human breast cancer. Correlation of relapse and survival with amplification of the Her-2/neu oncogene. *Science* **235:** 177–182

Van Slooten HJ, Bonsing BA, Hiller AJ, Colbern GT, Van Dierendonck JH, Cornelisse CJ and Smith HS (1995) Outgrowth of BT-474 human breast cancer cells in immune-deficient mice: a new in vivo model for hormone-dependent breast cancer. *Br J Cancer* **72:** 22–30

Watanabe M, Tanaka H, Kamada M, Okano JH, Takahishi H, Uchida K, Iwamura A, Zeniya M and Ohno T (1992) Establishment of the human BSMZ breast cancer cell line, which overexpresses the erbB-2 and c-myc genes. *Cancer Res* **52:** 5178–5182

Whitehead RH, Bertoncello I, Webber LM and Pedersen JS (1983) A new human breast carcinoma cell line (PMC42) with stem cell characteristics: I Morphologic characterization. *J Natl Cancer Inst* **70:** 649–661

Zhang RD, Fidler IJ and Price JE (1991) Relative malignant potential of human breast carcinoma cell lines established from pleural effusions and a brain metastasis. *Invas Metastas* **11:** 204–215

*© Cancer Research Campaign 1998*


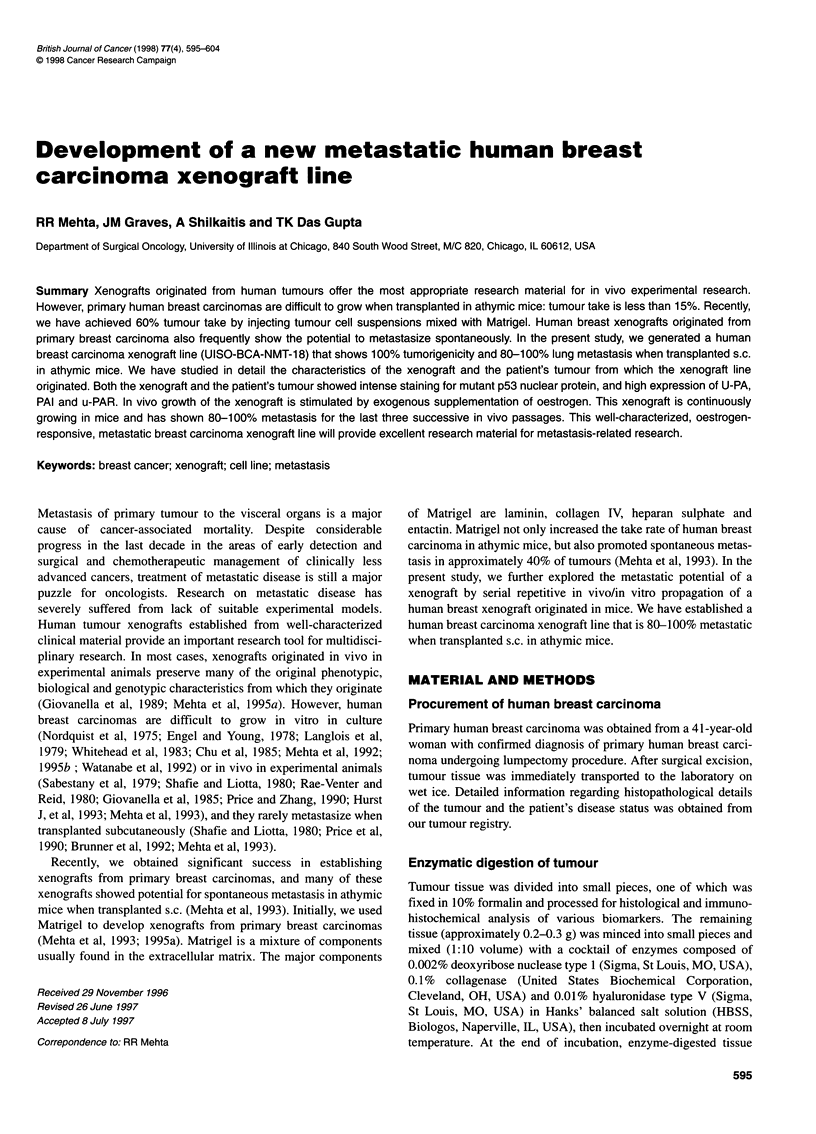

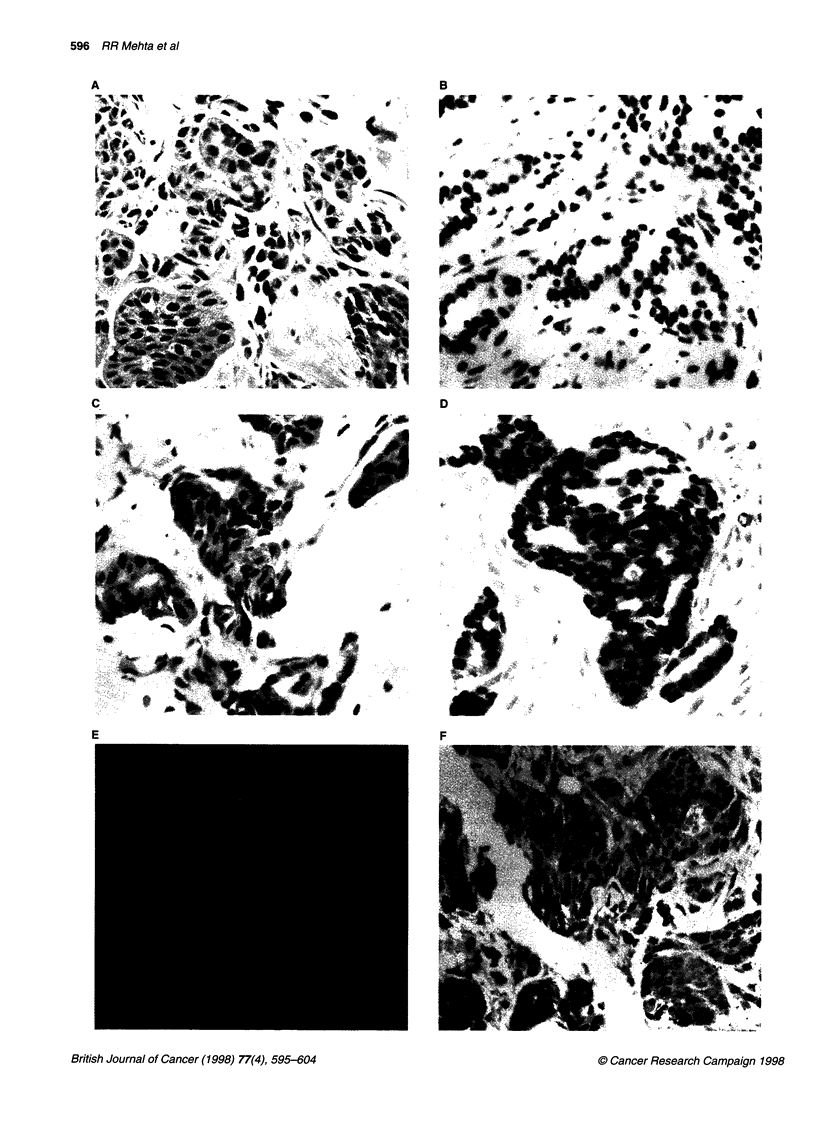

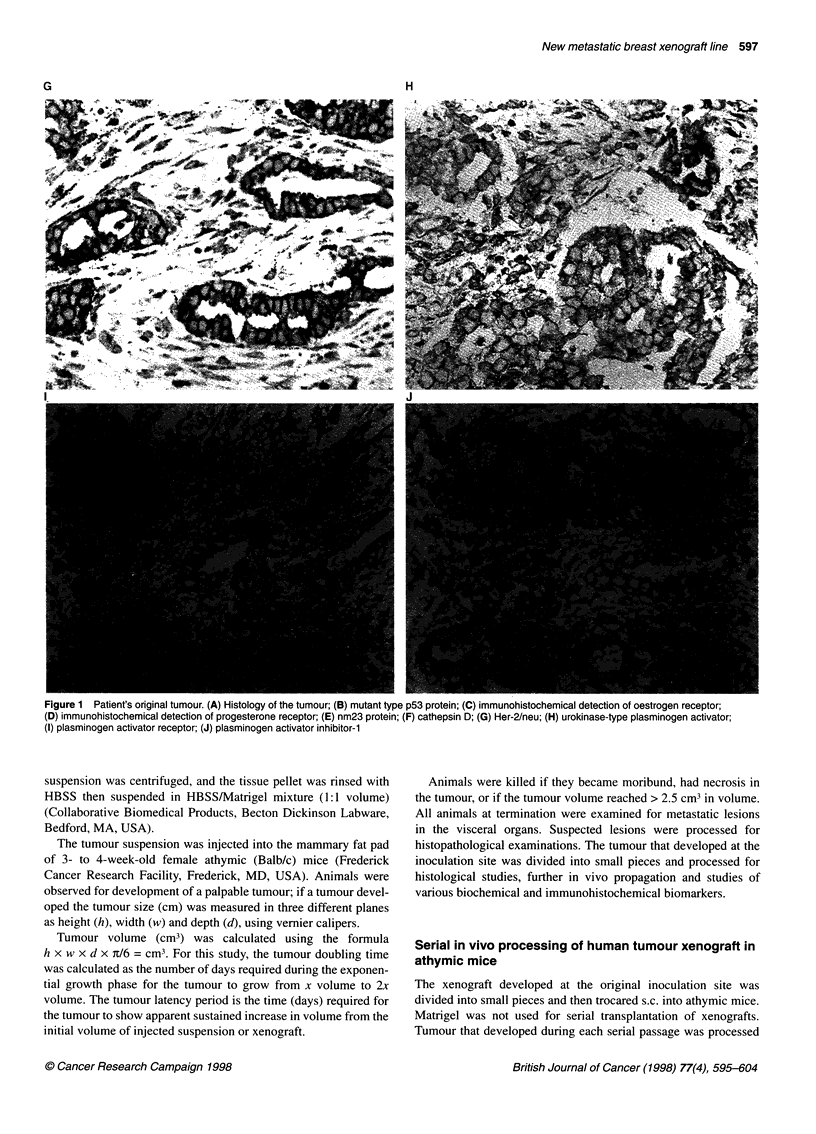

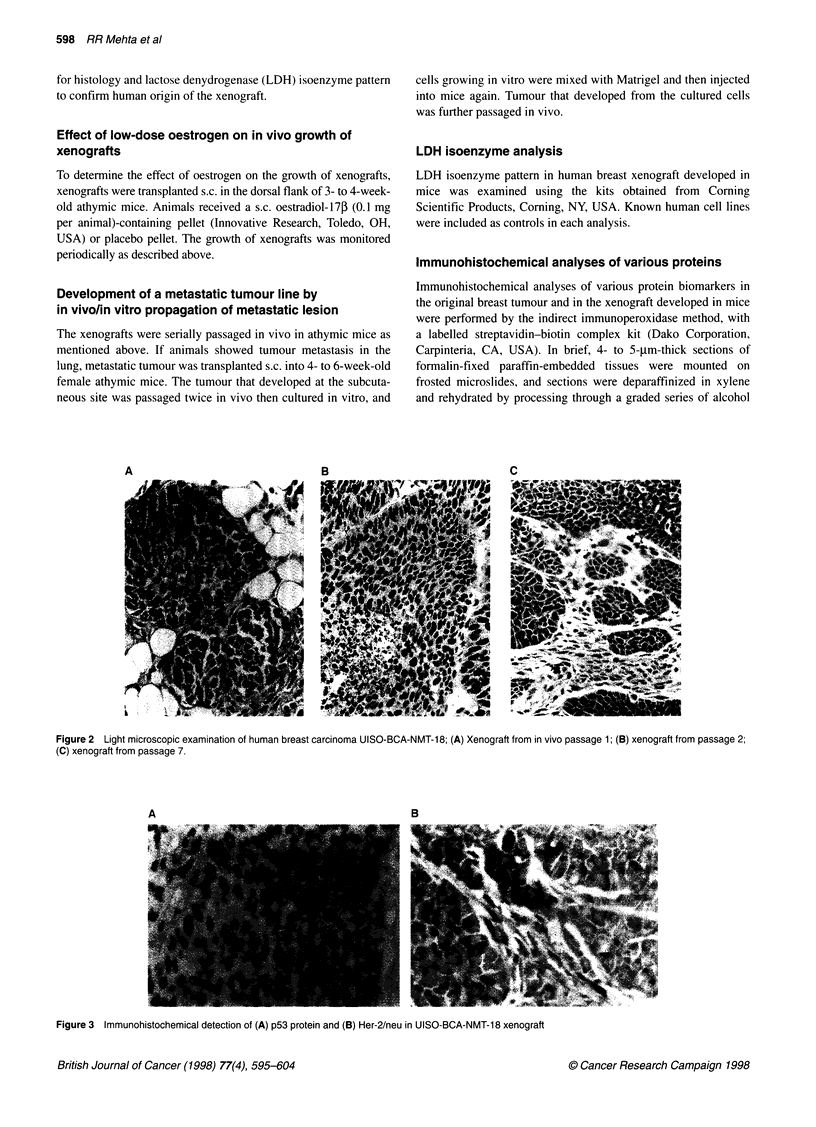

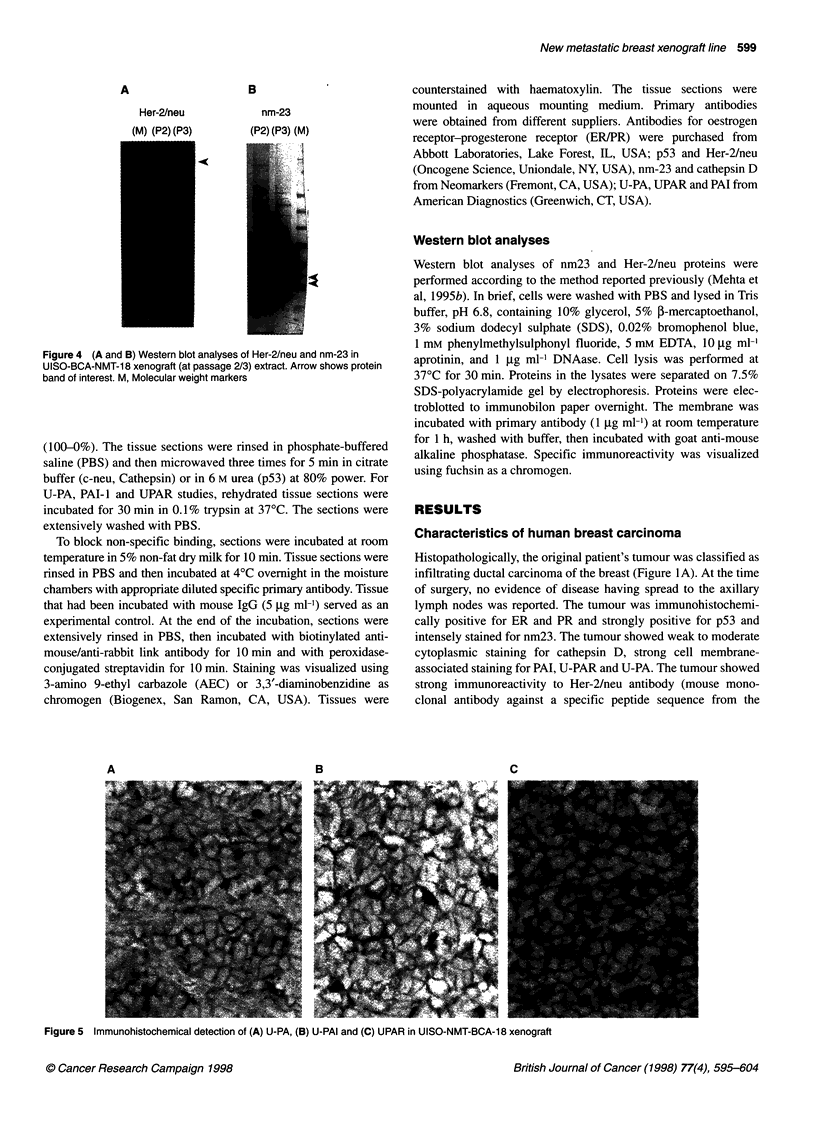

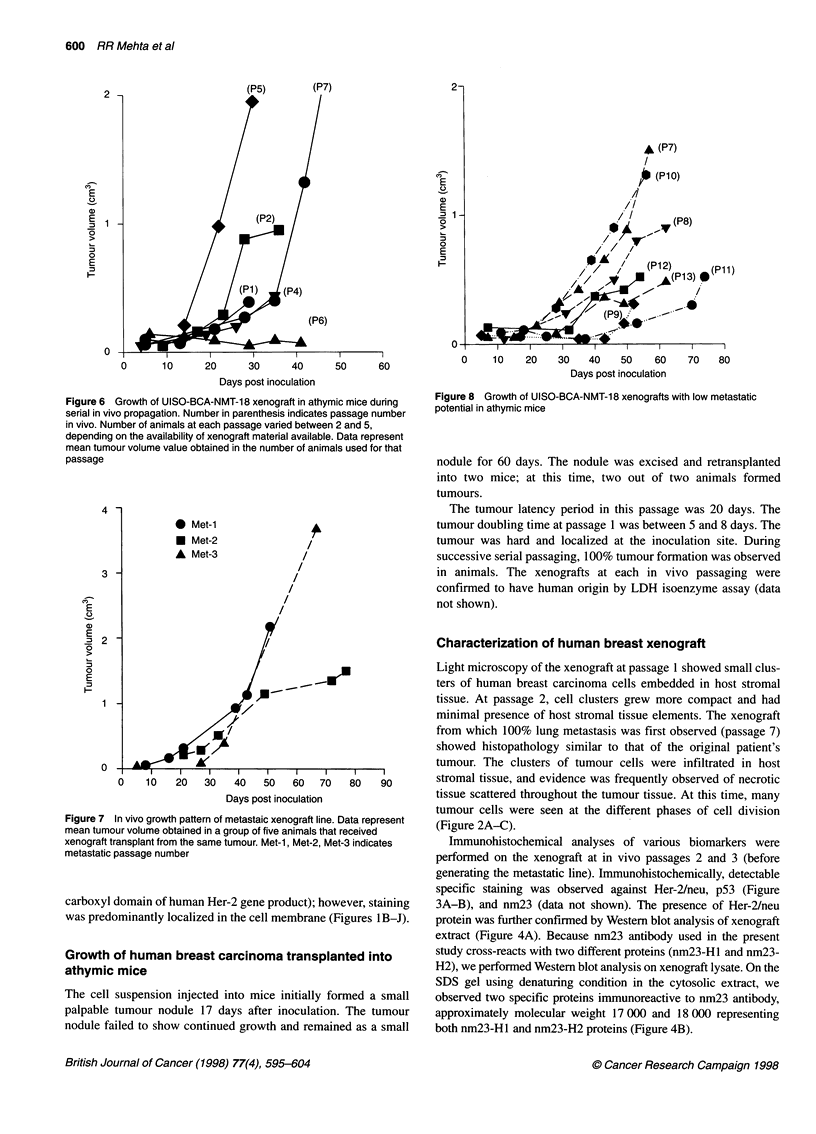

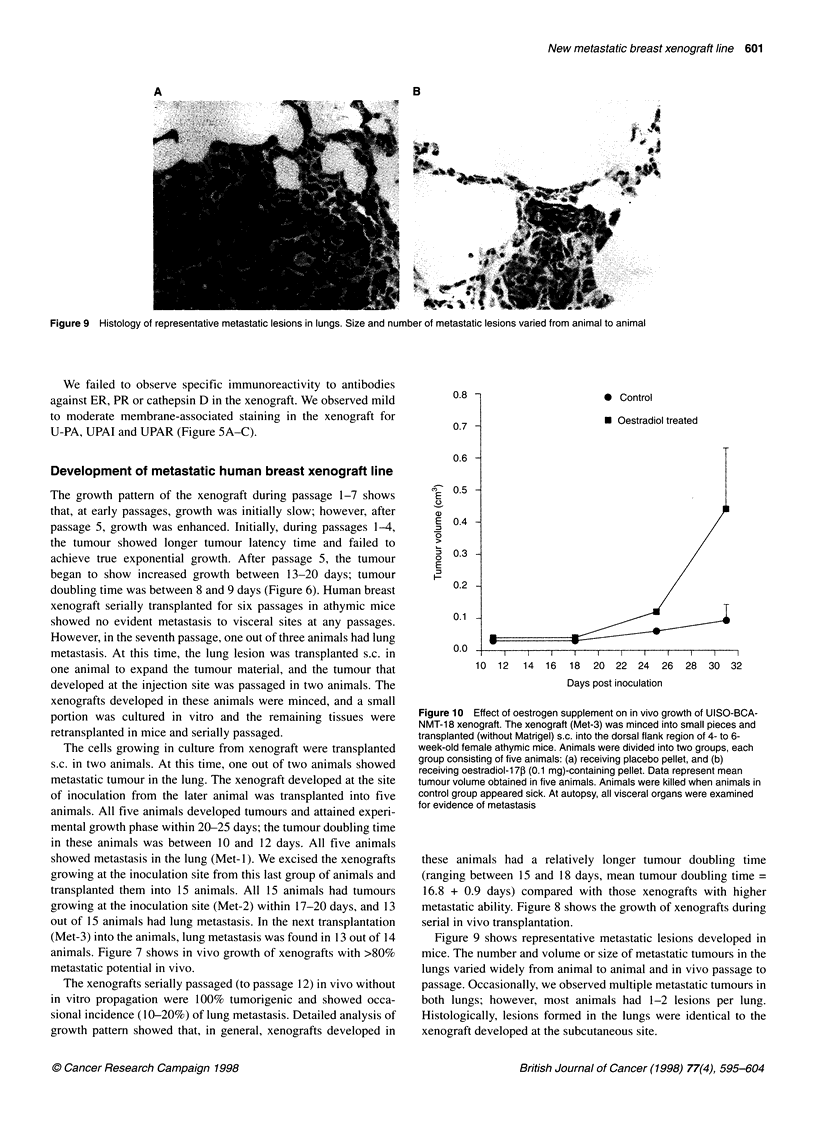

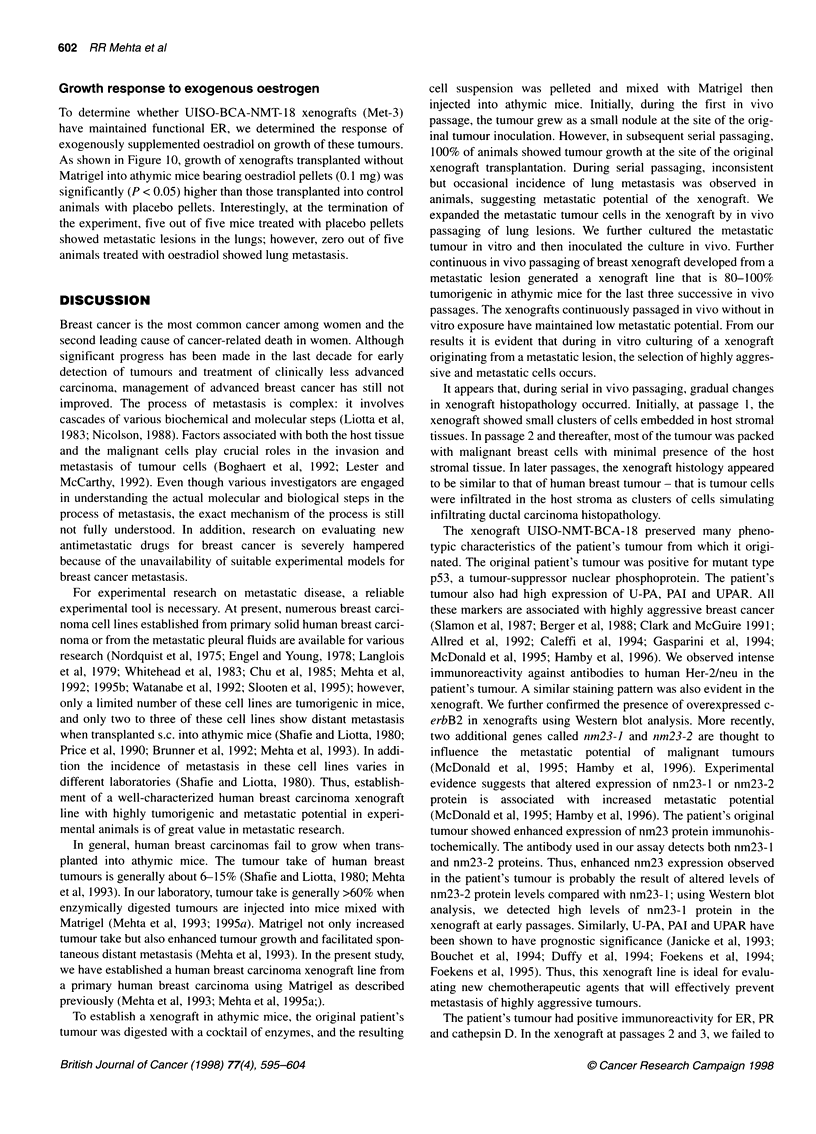

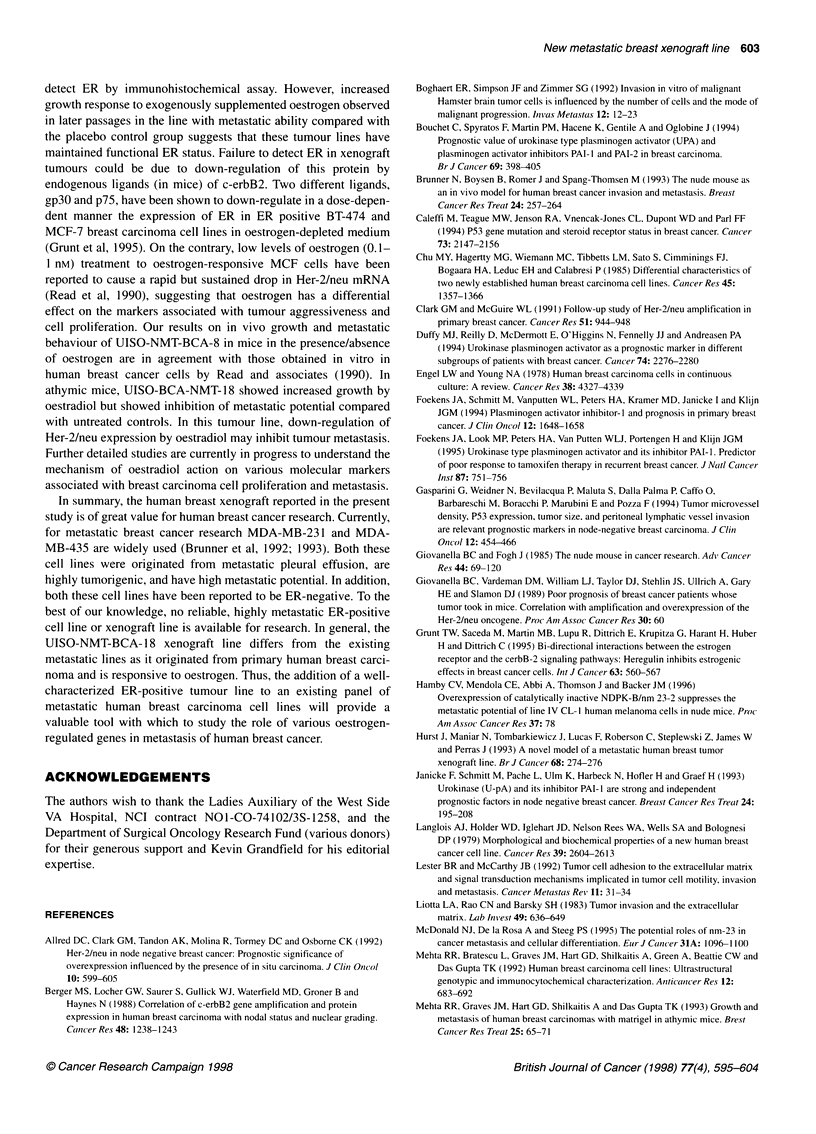

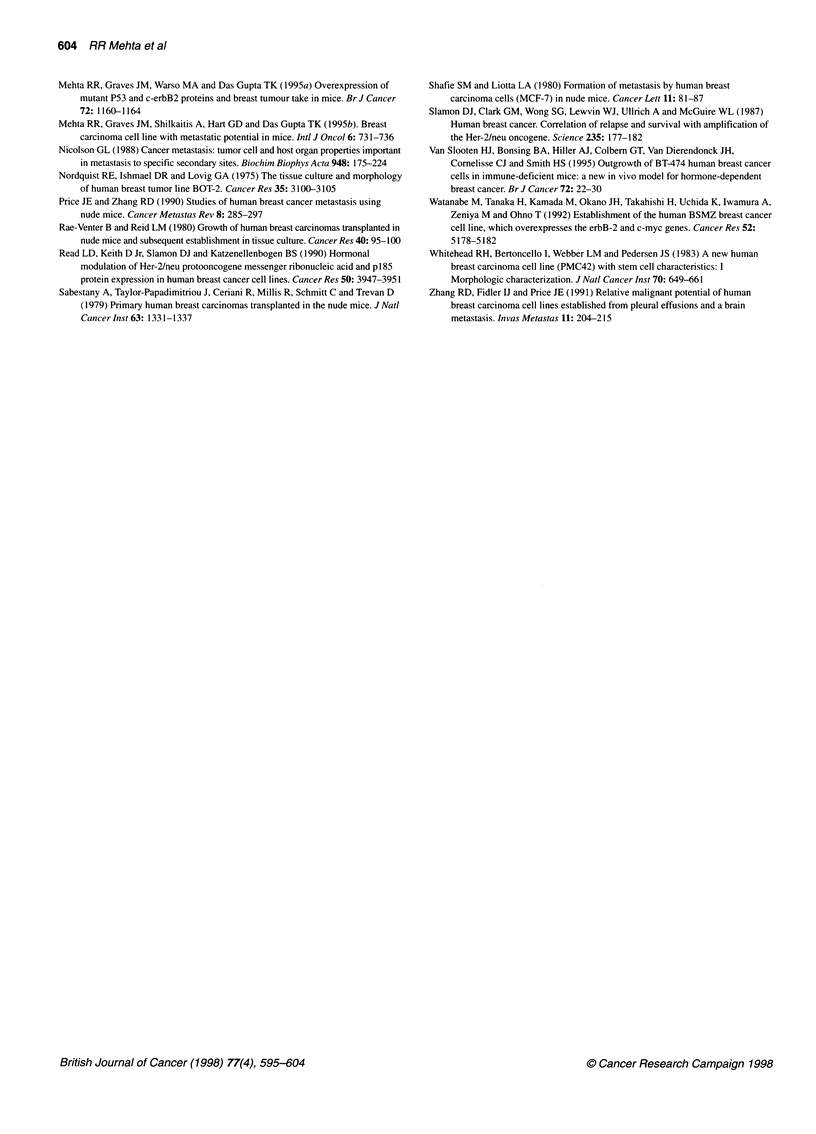


## References

[OCR_00814] Allred D. C., Clark G. M., Tandon A. K., Molina R., Tormey D. C., Osborne C. K., Gilchrist K. W., Mansour E. G., Abeloff M., Eudey L. (1992). HER-2/neu in node-negative breast cancer: prognostic significance of overexpression influenced by the presence of in situ carcinoma.. J Clin Oncol.

[OCR_00821] Berger M. S., Locher G. W., Saurer S., Gullick W. J., Waterfield M. D., Groner B., Hynes N. E. (1988). Correlation of c-erbB-2 gene amplification and protein expression in human breast carcinoma with nodal status and nuclear grading.. Cancer Res.

[OCR_00828] Boghaert E. R., Simpson J. F., Zimmer S. G. (1992). Invasion in vitro of malignant hamster brain tumor cells is influenced by the number of cells and the mode of malignant progression.. Invasion Metastasis.

[OCR_00833] Bouchet C., Spyratos F., Martin P. M., Hacène K., Gentile A., Oglobine J. (1994). Prognostic value of urokinase-type plasminogen activator (uPA) and plasminogen activator inhibitors PAI-1 and PAI-2 in breast carcinomas.. Br J Cancer.

[OCR_00840] Brünner N., Boysen B., Rømer J., Spang-Thomsen M. (1993). The nude mouse as an in vivo model for human breast cancer invasion and metastasis.. Breast Cancer Res Treat.

[OCR_00845] Caleffi M., Teague M. W., Jensen R. A., Vnencak-Jones C. L., Dupont W. D., Parl F. F. (1994). p53 gene mutations and steroid receptor status in breast cancer. Clinicopathologic correlations and prognostic assessment.. Cancer.

[OCR_00850] Chu M. Y., Hagerty M. G., Wiemann M. C., Tibbetts L. M., Sato S., Cummings F. J., Bogaars H. A., Leduc E. H., Calabresi P. (1985). Differential characteristics of two newly established human breast carcinoma cell lines.. Cancer Res.

[OCR_00856] Clark G. M., McGuire W. L. (1991). Follow-up study of HER-2/neu amplification in primary breast cancer.. Cancer Res.

[OCR_00860] Duffy M. J., Reilly D., McDermott E., O'Higgins N., Fennelly J. J., Andreasen P. A. (1994). Urokinase plasminogen activator as a prognostic marker in different subgroups of patients with breast cancer.. Cancer.

[OCR_00865] Engel L. W., Young N. A. (1978). Human breast carcinoma cells in continuous culture: a review.. Cancer Res.

[OCR_00876] Foekens J. A., Look M. P., Peters H. A., van Putten W. L., Portengen H., Klijn J. G. (1995). Urokinase-type plasminogen activator and its inhibitor PAI-1: predictors of poor response to tamoxifen therapy in recurrent breast cancer.. J Natl Cancer Inst.

[OCR_00869] Foekens J. A., Schmitt M., van Putten W. L., Peters H. A., Kramer M. D., Jänicke F., Klijn J. G. (1994). Plasminogen activator inhibitor-1 and prognosis in primary breast cancer.. J Clin Oncol.

[OCR_00880] Gasparini G., Weidner N., Bevilacqua P., Maluta S., Dalla Palma P., Caffo O., Barbareschi M., Boracchi P., Marubini E., Pozza F. (1994). Tumor microvessel density, p53 expression, tumor size, and peritumoral lymphatic vessel invasion are relevant prognostic markers in node-negative breast carcinoma.. J Clin Oncol.

[OCR_00887] Giovanella B. C., Fogh J. (1985). The nude mouse in cancer research.. Adv Cancer Res.

[OCR_00898] Grunt T. W., Saceda M., Martin M. B., Lupu R., Dittrich E., Krupitza G., Harant H., Huber H., Dittrich C. (1995). Bidirectional interactions between the estrogen receptor and the cerbB-2 signaling pathways: heregulin inhibits estrogenic effects in breast cancer cells.. Int J Cancer.

[OCR_00912] Hurst J., Maniar N., Tombarkiewicz J., Lucas F., Roberson C., Steplewski Z., James W., Perras J. (1993). A novel model of a metastatic human breast tumour xenograft line.. Br J Cancer.

[OCR_00917] Jänicke F., Schmitt M., Pache L., Ulm K., Harbeck N., Höfler H., Graeff H. (1993). Urokinase (uPA) and its inhibitor PAI-1 are strong and independent prognostic factors in node-negative breast cancer.. Breast Cancer Res Treat.

[OCR_00924] Langlois A. J., Holder W. D., Iglehart J. D., Nelson-Rees W. A., Wells S. A., Bolognesi D. P. (1979). Morphological and biochemical properties of a new human breast cancer cell line.. Cancer Res.

[OCR_00934] Liotta L. A., Rao C. N., Barsky S. H. (1983). Tumor invasion and the extracellular matrix.. Lab Invest.

[OCR_00938] MacDonald N. J., de la Rosa A., Steeg P. S. (1995). The potential roles of nm23 in cancer metastasis and cellular differentiation.. Eur J Cancer.

[OCR_00942] Mehta R. R., Bratescu L., Graves J. M., Hart G. D., Shilkaitis A., Green A., Beattie C. W., Das Gupta T. K. (1992). Human breast carcinoma cell lines: ultrastructural, genotypic, and immunocytochemical characterization.. Anticancer Res.

[OCR_00948] Mehta R. R., Graves J. M., Hart G. D., Shilkaitis A., Das Gupta T. K. (1993). Growth and metastasis of human breast carcinomas with Matrigel in athymic mice.. Breast Cancer Res Treat.

[OCR_00957] Mehta R. R., Graves J. M., Warso M. A., Das Gupta T. K. (1995). Overexpression of mutant p53 and c-erbB-2 proteins and breast tumour take in mice.. Br J Cancer.

[OCR_00966] Nicolson G. L. (1988). Cancer metastasis: tumor cell and host organ properties important in metastasis to specific secondary sites.. Biochim Biophys Acta.

[OCR_00970] Nordquist R. E., Ishmael D. R., Lovig C. A., Hyder D. M., Hoge A. F. (1975). The tissue culture and morphology of human breast tumor cell line BOT-2.. Cancer Res.

[OCR_00974] Price J. E., Zhang R. D. (1990). Studies of human breast cancer metastasis using nude mice.. Cancer Metastasis Rev.

[OCR_00978] Rae-Venter B., Reid L. M. (1980). Growth of human breast carcinomas in nude mice and subsequent establishment in tissue culture.. Cancer Res.

[OCR_00981] Read L. D., Keith D., Slamon D. J., Katzenellenbogen B. S. (1990). Hormonal modulation of HER-2/neu protooncogene messenger ribonucleic acid and p185 protein expression in human breast cancer cell lines.. Cancer Res.

[OCR_00995] Slamon D. J., Clark G. M., Wong S. G., Levin W. J., Ullrich A., McGuire W. L. (1987). Human breast cancer: correlation of relapse and survival with amplification of the HER-2/neu oncogene.. Science.

[OCR_01006] Watanabe M., Tanaka H., Kamada M., Okano J. H., Takahashi H., Uchida K., Iwamura A., Zeniya M., Ohno T. (1992). Establishment of the human BSMZ breast cancer cell line, which overexpresses the erbB-2 and c-myc genes.. Cancer Res.

[OCR_01012] Whitehead R. H., Bertoncello I., Webber L. M., Pedersen J. S. (1983). A new human breast carcinoma cell line (PMC42) with stem cell characteristics. I. Morphologic characterization.. J Natl Cancer Inst.

[OCR_01017] Zhang R. D., Fidler I. J., Price J. E. (1991). Relative malignant potential of human breast carcinoma cell lines established from pleural effusions and a brain metastasis.. Invasion Metastasis.

[OCR_01000] van Slooten H. J., Bonsing B. A., Hiller A. J., Colbern G. T., van Dierendonck J. H., Cornelisse C. J., Smith H. S. (1995). Outgrowth of BT-474 human breast cancer cells in immune-deficient mice: a new in vivo model for hormone-dependent breast cancer.. Br J Cancer.

